# Aggregation tendencies in the p53 family are modulated by backbone hydrogen bonds

**DOI:** 10.1038/srep32535

**Published:** 2016-09-07

**Authors:** Elio A. Cino, Iaci N. Soares, Murilo M. Pedrote, Guilherme A. P. de Oliveira, Jerson L. Silva

**Affiliations:** 1 Programa de Biologia Estrutural, Instituto de Bioquímica Médica Leopoldo de Meis, Instituto Nacional de Biologia Estrutural e Bioimagem, Universidade Federal do Rio de Janeiro, Rio de Janeiro, 21941-902, RJ, Brazil

## Abstract

The p53 family of proteins is comprised of p53, p63 and p73. Because the p53 DNA binding domain (DBD) is naturally unstable and possesses an amyloidogenic sequence, it is prone to form amyloid fibrils, causing loss of functions. To develop p53 therapies, it is necessary to understand the molecular basis of p53 instability and aggregation. Light scattering, thioflavin T (ThT) and high hydrostatic pressure (HHP) assays showed that p53 DBD aggregates faster and to a greater extent than p63 and p73 DBDs, and was more susceptible to denaturation. The aggregation tendencies of p53, p63, and p73 DBDs were strongly correlated with their thermal stabilities. Molecular Dynamics (MD) simulations indicated specific regions of structural heterogeneity unique to p53, which may be promoted by elevated incidence of exposed backbone hydrogen bonds (BHBs). The results indicate regions of structural vulnerability in the p53 DBD, suggesting new targetable sites for modulating p53 stability and aggregation, a potential approach to cancer therapy.

The p53 family of proteins comprises p53, p63, and p73 transcriptional factors^1^. Although most invertebrates have only a p63/p73 like gene, duplication events around the evolution of cartilaginous and bony vertebrates produced the three family members^2^. A primary role of the ancestral protein is to protect germ line cells from DNA damage–a function that has been preserved for over a billion years^2^. Of the three proteins, p53 is the most evolutionarily divergent from its ancestral version, as it has taken on new tumor suppressor roles in protecting somatic stem cells from DNA damage^2^. In contrast, p63 and p73 have diverged comparably less from the ancestral protein^3^.

The divergent evolution of p53 family members is evident from their sequences. p53 is considerably shorter than p63/p73, and lacks several C-terminal domains that confer unique functions to p63/p73^4^. p63 and p73 are of similar length, and have all major domains in common. The region with the highest similarity among the three members is the DNA binding domain (DBD), which is responsible for recognizing and binding to target gene sequences. The DBDs share ~60% identity across the family, while p63 and p73 DBDs are ~85% identical^4^. Comparison of the number of amino acids changes in p53 family DBD sequences from different vertebrates shows p53 evolving the fastest, followed by p73, and then p63^2^.

As the p53 DBD has evolved more rapidly in order to assume distinct functions, it has become considerably less stable than the DBDs of p63 and p73^5^. Levels of p53 in cells must be tightly regulated for it to function properly. When compared to p63 and p73, p53 has a much shorter half-life, indicating that the p53 DBD has evolved to be only as stable as necessary to function at typical body temperatures^6^. p53 is the most commonly mutated protein found in cancers, and over 90% of p53 mutations occur in its DBD^6^. A large number of DBD mutations cause structural destabilization of the already labile DBD, making them prone to unfold at 37 °C^6^.

Structurally destabilizing DBD mutations can lead to loss of p53 function and dominant negative effect on wt p53 through two different mechanisms. In cells, p53 exists as an assortment of monomers, dimers and tetramers^7^. Tetramers of unstable DBD mutants, or heterotetramers of wt and mutants fail to efficiently bind to DNA, impairing tumor suppressor functions^8^. In the second mechanism, p53 inactivation occurs when molecules of unfolded DBD exposing an aggregation prone sequence (residues 251–257) self-aggregate, or coaggregate with molecules of unfolded wt p53^9^,^10^. p53 aggregation kinetics shows two distinct processes: A relatively slow generation of an aggregation competent state, which refers to full or partial unfolding of the DBD to expose the aggregation nucleus, followed by a rapid aggregation phase^11^. Wt p53 DBD has a tendency to form fibrillar aggregates; however, structurally destabilizing mutants contain a higher population of unfolded molecules in solution, and can have substantially higher aggregation rates^12^. Biophysical studies on p53 aggregates have found they have amyloid-like properties, such as β-rich structure, water excluded cavities, and ability to bind the fluorescent amyloid marker ThT^12^,^13^. Cell-to-cell transmission of p53 DBD and aggregates has been demonstrated^14^,^15^. Spontaneous aggregation of p53 into typical amyloid structures that can be transmitted to other cells is consistent with prion-like behavior^16^. Amyloid aggregates of mutant p53 have been identified in tissues from different tumors, such as breast cancer^12^, and malignant skin tumors^17^.

Despite p63 and p73 having aggregation nucleating sequences similar to p53 within their DBDs, and ability to coaggregate with some p53 DBD mutants, causing impairment of their functions, recent data suggests that they have much lower aggregation tendencies, and that relatively few p63/p73 DBDs are incorporated into p53 aggregates^10^. The behavior is likely in part due to their DBDs being more stable compared to that of p53^18^. Consistent with this idea is the lower aggregation tendencies of enhanced stability p53 DBDs. The quadruple mutant (QM) p53 DBD (residues 94–312), has a thermal melting temperature 5.6 °C higher than wt p53 DBD, and exhibits slower aggregation kinetics^19^,^20^. N-terminal extension of the p53 QM DBD by a few amino acids (starting at residue 89 rather than 94) increases thermal stability by an additional 2 °C, and further reduces the rate of aggregation relative to p53 QM DBD^21^. In addition to enhancing DBD stability by sequence modifications, there are also efforts to find small molecules that can stabilize p53. The decreased stability of the oncogenic Y220C mutant can be rescued with several compounds, which can also reduce the elevated aggregation rate of this mutant^22^.

The relationship between DBD stability and tendency to aggregate provides some insights for assessing how aggregation prone a given DBD construct might be, but it does not reveal the underlying molecular features. Here, differential aggregation propensities and amyloid formation in the p53 family are assessed using customary experimental methodology, and the molecular details of different stabilities and aggregation characteristics are analyzed using long timescale MD simulations. Aggregation assays showed that p53 aggregated faster and to a greater extent than p73, whereas p63 showed negligible aggregation. The aggregation tendencies of p53, p63, and p73 DBDs were strongly correlated with their thermal stabilities. In agreement with the kinetic studies, when challenged by hydrostatic pressure, p53 family members revealed different susceptibilities to unfolding and aggregation (p53 wt > p53 QM > p73 > p63). MD simulations showed that distinct regions of p53 deviate substantially more from its initial structure, and show elevated incidence of exposed BHBs compared to p63 and p73. The results indicate regions of p53 that may be prone to structural instability, and provide molecular-level insights into the causes.

## Results

### p53 DBD aggregates considerably more than p63 and p73 DBDs *in vitro*

**Figure 1:**
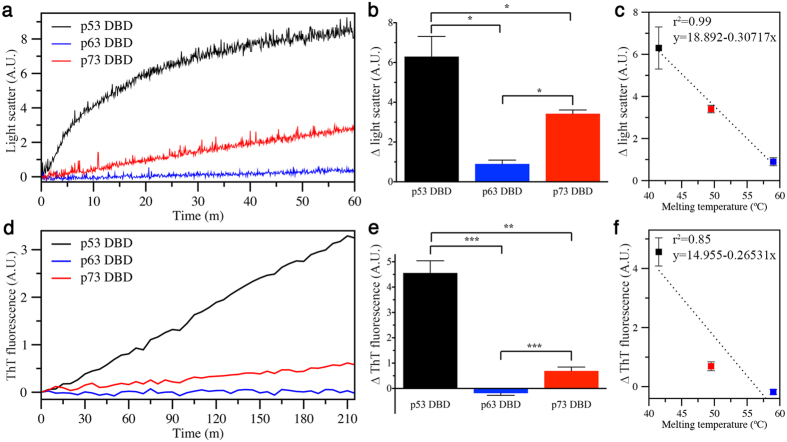
Different aggregation propensities of p53 family DBDs. (**a**) Representative light scattering experiment at 500 nm. (**b**) Average and standard errors (N = 4) of the change in light scattering intensity between the initial and final readings. Statistical significance was estimated by Mann-Whitney test (*p < 0.05). (**c**) Pearson correlation of thermal melting values of the DBDs^18^ and the ∆ light scattering avg. ± s.e. values. (**d**) Representative ThT fluorescence experiment. (**e**) Average and standard errors (N = 4) of the change in ThT fluorescence between the initial and final readings. Statistical significance was estimated by Mann-Whitney test (**p < 0.005; ***p < 0.0005). (**f**) Pearson correlation of thermal melting values of the DBDs and the ∆ ThT fluorescence avg. ± s.e. values.

Light scattering and ThT fluorescence measurements were performed on purified DBDs of wt p53, p63, and p73 at 37 °C to compare their aggregation characteristics. p53 showed a short lag phase, plateauing at intensities ~2–3 fold higher than p73, while p63 readings were negligible (Fig. 1a,b). The differences in light scattering intensities between the three DBDs were statistically significant, and strongly correlated with their melting temperatures (Fig. 1b,c). ThT binding assays were performed to assess amyloid aggregate formation at 37 °C. Maximum ThT fluorescence values for p53 were ~6-fold higher than p73 (Fig. 1d,e). p63 samples did not yield detectable fluorescence. The three DBDs showed statistically significant differences in ThT fluorescence, which were also strongly correlated with their thermal melting temperatures (Fig. 1e,f).

### Denaturation and aggregation susceptibilities upon pressure

**Figure 2:**
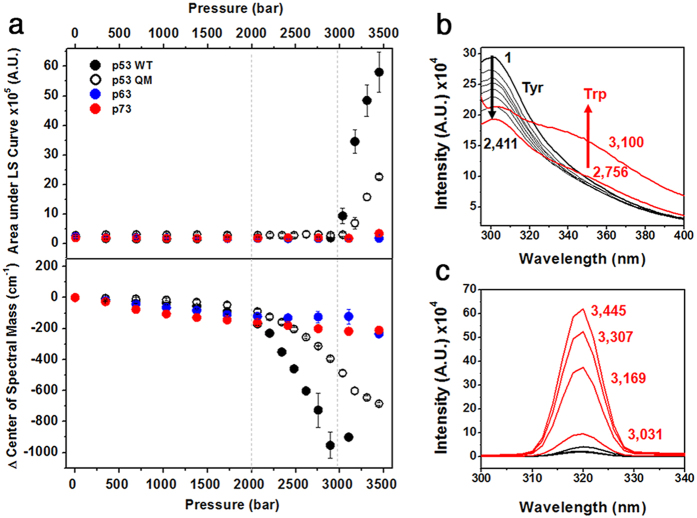
Stability of p53 family members against pressure. (**a**) Light scattering (top) and changes in the center of spectral mass from Tyr/Trp emission spectra (bottom) were recorded as a function of increasing pressures for p53 family members, and p53 QM. Vertical dashed lines show the pressure range in which initial unfolding but not aggregation takes place. (**b**) Tyr/Trp emission spectra and (**c**) light scattering dependence upon pressure increments for p53 wt. Inset values represent pressure in bar units.

HHP is a commonly applied technique to characterize folding intermediates and study protein aggregation^23^,^24^. Correlation between the population of folding/unfolding intermediates, and amyloid aggregation of HHP-perturbed wt p53 DBD and the R248Q mutant has been demonstrated^25^. As an additional approach to test the stability and aggregation susceptibility of p53 family members, the changes in the fluorescence emission of Tyr/Trp residues and light scattering were monitored at 25 °C under different pressures. The HHP light scattering profiles (Fig. 2a, top) were similar to the measurements at 37 °C (Fig. 1), showing p53wt > p73 > p63. At pressures above 2 kbar, wt and QM p53 began to unfold, whereas p63 and p73 maintained their structural integrity (Fig. 2a, bottom). Although the p53 QM yielded lower light scattering intensities and delayed pressure-induced denaturation compared to wt p53, it was still considerably more susceptible to aggregation and unfolding than p63/p73. The Tyr/Trp fluorescence and light scattering data provides information about the aggregation-prone intermediate conformation of p53. Prior to aggregation, the HHP-induced protein denaturation (2 to 3 kbar) leads p53 to populate an aggregation-prone conformation in which Tyr residues become partially exposed to the solvent, but the single Trp is still relatively buried. The HHP results suggest that p53 DBD aggregation starts concomitantly with the exposure of the single Trp to the solvent (Fig. 2b,c).

### Sequence and structure comparison of p53 family DBDs

**Figure 3:**
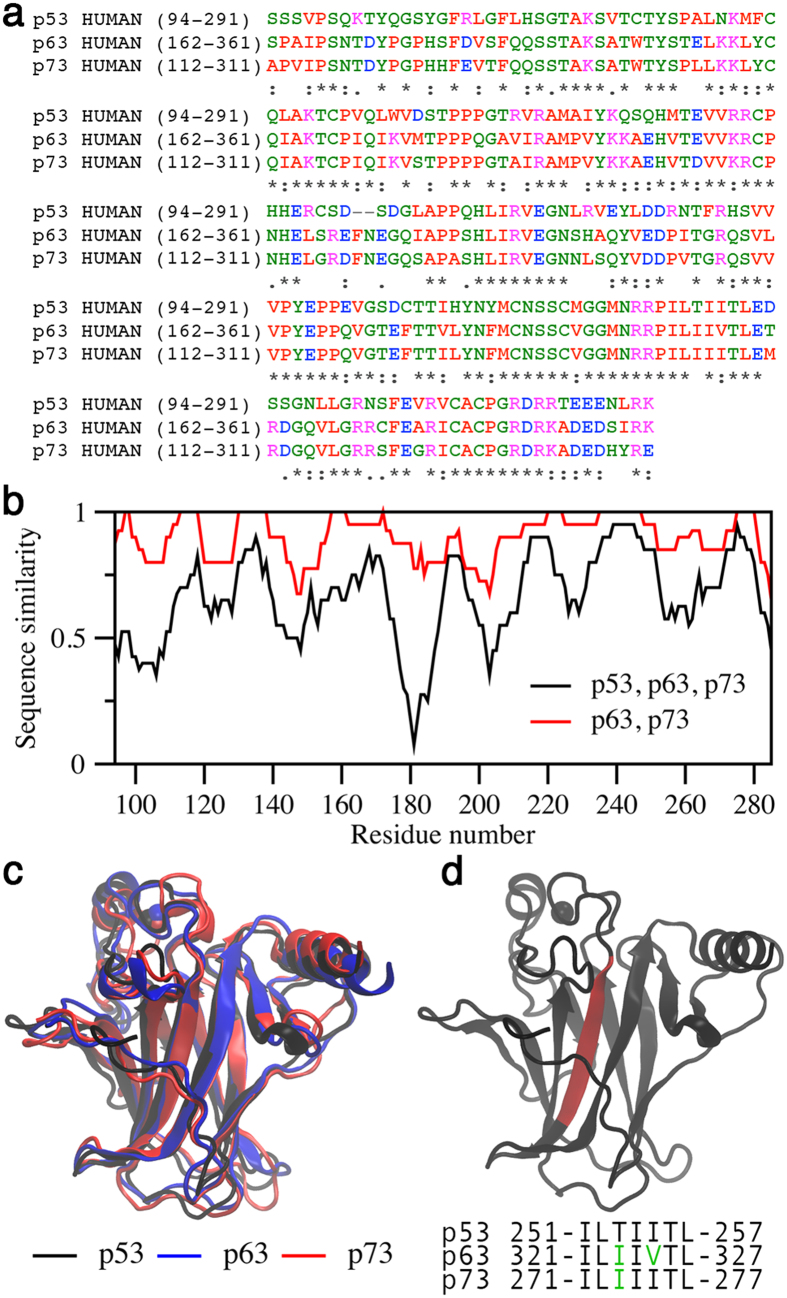
Comparison of the sequences and structures of p53 family DBDs. (**a**) Clustal Omega multiple sequence alignment of p53, p63 and p73 DBDs^28^. The default Clustal Omega coloring scheme was employed (hydrophobic, red; acidic, blue; basic, magenta; polar, green). (**b**) Quantification of sequence similarity based upon the gapped alignment of the DBD sequences, considering fully conserved (*), highly conserved (:), moderately conserved (.), non-conserved () and gapped positions (-). (**c**) Superposition of DBD structures (p53, black, 2FEJ; p63, blue, 2RMN; p73, red, 2XWC)^30^,^33^. (**d**) (top) p53 DBD structure showing the location of the aggregation prone region (red), and (bottom) PASTA predicted regions of amyloid aggregation in the p53 family DBDs^26^.

Quantitative comparison of DBD sequences of p53 family members indicates several regions where p53 differs from p63 and p73 (Fig. 3a,b). Sequence similarity between p63 and p73 DBDs is ~90%, dropping to 66% when p53 is included. The region spanning 176–188, which corresponds to helix 1 (H1), shows the greatest extent of sequence deviation between p53 and p63/p73. In addition to lower sequence identity, the p53 alignment contains two gaps in this segment. Despite these differences, the DBDs of the three family members are structurally alike with low Cα rmsds of ~0.2 nm (Fig. 3c). The PASTA 2.0 algorithm^26^, which predicts amyloidogenic amino acid sequences, identified a single 7-residue segment of corresponding positions to be the only aggregation prone region (Fig. 3d). The identified segment matches the previously reported aggregation nucleus of p53^27^.

### Structural integrity of p53 family DBDs

**Figure 4:**
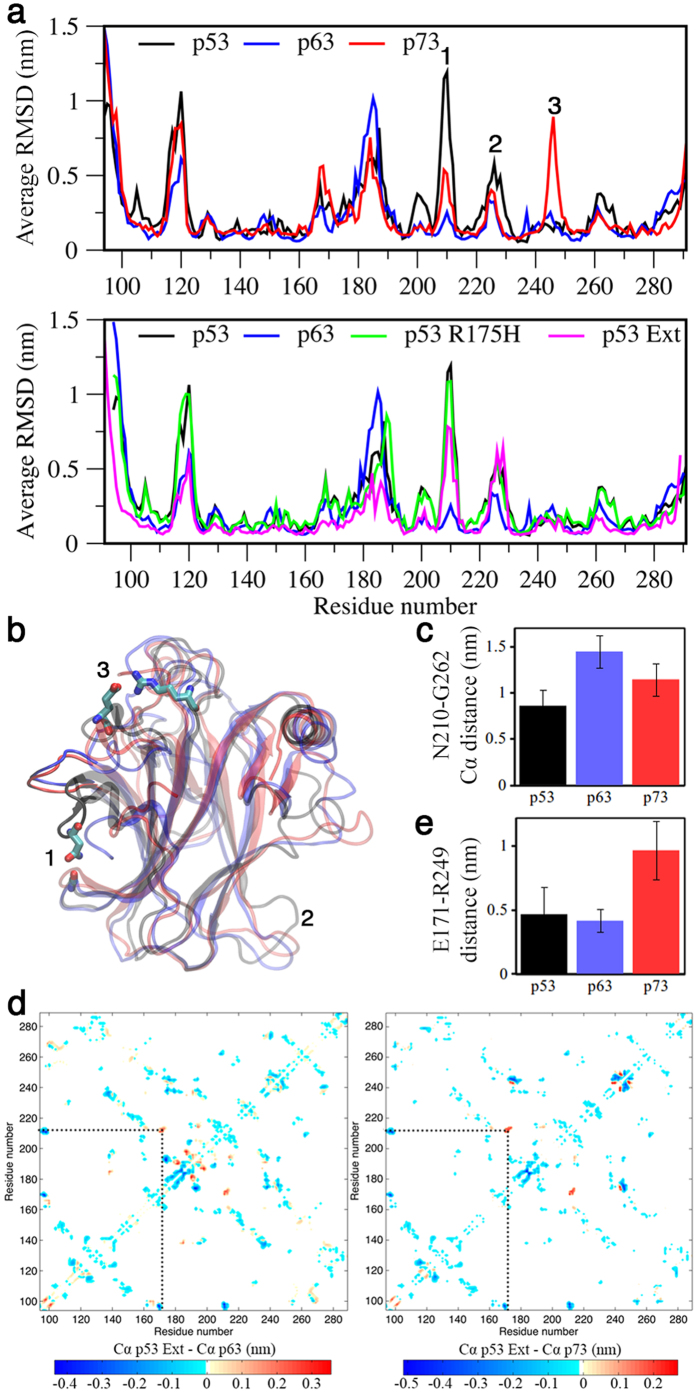
Residue-specific deviations from the initial structures. (**a**) Average RMSD from the starting structures. (**b**) Representative structures from the largest clusters, illustrating the major areas of RMSD difference. Clustering analysis was performed using the g_cluster single-linkage algorithm^62^,^68^, with a 0.15 nm Cα rmsd cut-off. Residues E171, R175, N210 and G262 of p53 are displayed in atomic detail for reference. (**c**) Average p53 wt N210-G262 Cα distance, and corresponding p63 and p73 distances (I280-G332 and V230-G282, respectively). (**d**) Subtraction of Cα-Cα minimum distances matrices (p53 Ext minus p63, left and p73, right), of interatomic contacts <1.0 nm. Dashed line indicates the E171-N210 pair distance. (**e**) Average distances between E171 (center of mass of side chain oxygen atoms) and R249 (center of mass of side chain nitrogen atoms) of p53 wt, and the corresponding residue pairs from p63 (E239-R319), and p73 (D189-R269).

Long timescale MD simulations were carried out in order to evaluate the roles of protein dynamics on the stabilities and aggregation properties of the DBDs. In addition to p53, p63, and p73 DBD constructs (Fig. 3a), a p53 R175H mutant and an N-terminally extended structure (p53 Ext) were also simulated to assess the apparent higher, and lower aggregation rates, respectively, of these variants. The residue-specific Cα RMSDs from the initial structures were used to identify regions prone to structural variation (Fig. 4a). Wt p53 and the R175H mutant exhibited the highest average RMSD per residue (0.27 ± 0.08 and 0.27 ± 0.04, respectively), followed by p73 (0.24 ± 0.01), p63 (0.22 ± 0.02), and p53 Ext (0.18 ± 0.02) (Supplementary Table 1). The fraction of native contacts were consistent with the RMSDs: p53 Ext had the highest amount (78%), p63 and p73 had ~10% less, while p53 and p53 R175H had ~60%.

Despite p53 Ext having lower overall deviation from its initial structure compared to p63 and p73, the aggregation of this construct, even when combined with additional stabilizing mutations, is not completely abolished^21^. Therefore, in order to concentrate on the features which most likely to cause p53 aggregation, we compare p53 Ext with p63 and p73 in some of the subsequent analysis. Two regions around residues 207–213 (S6/S7 turn, Fig. 4a, label 1) and 220–230 (Fig. 4a, label 2) of p53 Ext show elevated deviations compared with p63. Mapping of these differences onto representative structures from the largest clusters illustrates the different conformation of the S6/S7 turn, and of the loop around Y220 (Fig. 4b). To quantitatively illustrate the differences in the S6/S7 turn, average distances between N210 and G262 Cα atoms were measured, showing the closer approach of these atoms in p53, relative to the corresponding pairs in p63 and p73 (Fig. 4c). The different S6/S7 turn conformation can also be identified by subtraction of minimum Cα-Cα distance matrices (Fig. 4d). The p63 or p73 distance matrix was subtracted from that of p53 Ext, resulting in difference matrices where negative values indicate closer Cα-Cα distances in p53, and positive values show pairs that are in closer contact in p63 or p73. Both of the difference matrices are dominated by negative values, indicating that p53 Ext had closer Cα-Cα pair distances overall compared to p63 and p73. However, both p63 and p73 matrices show clear positive values between positions corresponding contacts between residues around N210 and E171 (labels 1 and 3), illustrating the different S6/S7 conformation in p53 Ext. The analysis also shows contacts between W91 and R174 in p53 Ext (Fig. 4d), which are thought to be responsible for the higher stability of the p53 Ext construct^21^.

A region of high deviation was observed in p73, peaking around the position corresponding to R249 in p53 (Fig. 4a, label 3). Interestingly, p53 and p63 contain a native E171-R249 salt-bridge (Fig. 4b), which is an important interaction for p53 stability, as evidenced by higher aggregation rates of mutations at, and around position 249^29^. In p73, the residue corresponding to E171 is an aspartic acid, which is not able to approach R249 close enough to make a stable interaction (Fig. 4e). This is not obvious from the crystal structure of the p73 DBD, which places the sidechain functional groups ~0.45 nm apart^30^. p53 R175H had an RMSD profile similar to wt p53, but with moderately elevated values around residues 188–190 (Fig. 4a). The other physical properties that were compared, including secondary structure content, radius of gyration, the number of Cα-Cα contacts, intramolecular hydrogen bonds, and protein-water hydrogen bonds, were not considerably different among the different constructs (Supplementary Table 1).

### BHB defects in p53 DBD

**Figure 5:**
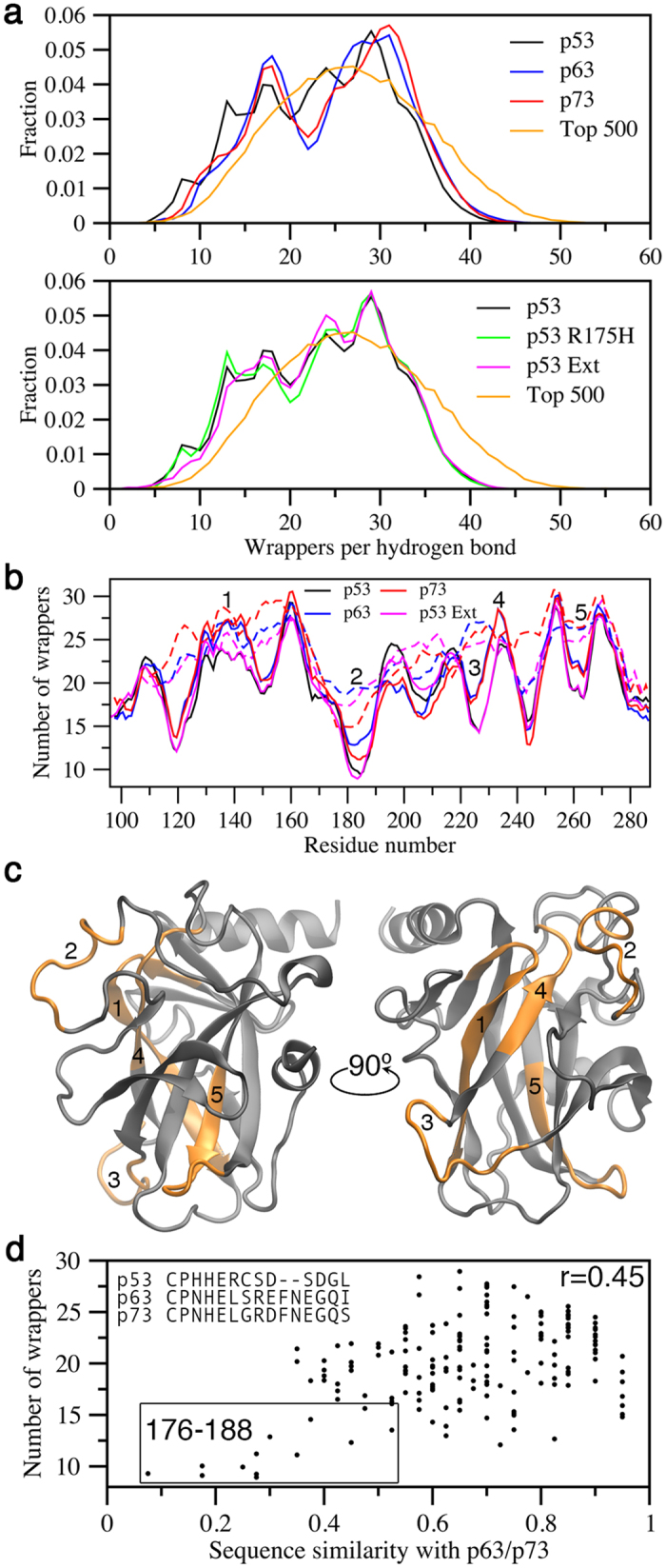
BHB protection in p53 family DBDs. (**a**) Distribution of the number of wrappers per hydrogen bond. (**b**) Number of protecting groups per residue (expressed as a 10-residue moving window average for clarity). Dashed lines indicate the protecting group analysis on the initial structures, before MD simulation. (**c**) Mapping of the major regions of differences, (**b**), onto the p53 DBD structure. (**d**) Correlation between sequence similarity (Fig. 3b) and number of BHB wrappers.

Protection of BHBs is a crucial property that is directly related to protein stability and tendency to form amyloid aggregates^31^. The absence of sufficient hydrophobic atoms around BHBs promotes interaction of the labile bond with water molecules, weakening backbone interactions, thus reducing structural integrity. Compared to the Top 500 PDB dataset^32^, BHBs in p53 family DBDs have lower numbers of protecting groups, which are commonly referred to as wrappers (Fig. 5a). The database structures contained an average of 17% of all BHBs in an underprotected state (less than 19 wrappers per BHB); meanwhile, the value was 30% for p53 and p53 R175H, 27% for p53 Ext, and 25% for p63 and p73. Also, on the right-hand side of the distribution, p63 and p73 show elevated numbers of well-protected BHBs compared to p53 constructs. The residue-specific view of BHB protection (Fig. 5b) reveals 5 regions where p53 and p53 Ext are less protected than p63/p73; however, only two of these regions (labels 2 and 3) have an average number of protecting groups below the cutoff for classification as an underprotected BHB. Interestingly, those two regions are not apparent from analysis of the initial structures used for the simulations, which show very similar protection patterns for p53 Ext and p63 (Fig. 5b). It is also notable that regions 1, 2, and 4, while discontinuous in sequence, are spatially proximal, forming a large surface patch that also makes contacts with region 5, which encompasses part of the aggregation nucleus (Fig. 5c). Per-residue BHB analysis of p73 yielded a similar pattern as p63, with the same 5 regions of difference to p53 easily identifiable; however, region 2 showed lower protection values than p63, but higher than p53. A moderate correlation exists between sequence similarity to p63/p73 and extent of BHB protection (Fig. 5d), with residues 176–188 showing the greatest sequence divergence and highest incidence of underprotected BHBs.

## Discussion

Over the last several decades, natural instability of p53 DBD, and its tendency to form amyloid-like aggregates has been established, and the molecular explanations of these properties and their relationship have begun to emerge. Because p53 is the most commonly mutated protein in cancer, with the majority of mutations occurring in the aggregation-prone DBD, it is a major therapeutic target. Design of stabilized p53 DBDs, and identification of potential small molecule binding sites are some of the most commonly explored strategies for restoring p53 functions^6^. Through experimental comparison of p53, p63, and p73 DBD aggregation and long timescale MD simulations, this work provides new molecular-level explanations of differential stabilities and aggregation tendencies in the p53 family.

Although all three p53 family members contain similar aggregation sequences within their DBDs, p53 is considerably more aggregation prone than the other members (Fig. 1). One notable difference in the aggregation sequences is position T253 in p53, which is an Ile in p63 and p73 (Fig. 3d). In p53, T253 is buried in the hydrophobic core, and shows suboptimal hydrogen bonding with Y236^33^. Double mutation (DM) of the T253/Y236 pair to the p63/p73 equivalent I253/F236 leads to moderate stability increases of 1.6 kcal/mol^33^. Combining the significantly stabilizing QM and moderately stabilizing DM mutations to generate a hexamutant (HM) p53 DBD only marginally increases the thermal denaturation temperature from 51.0 °C (QM) to 51.3 °C (HM)^34^. Based on our data (Fig. 1), the QM and HM constructs, which have ~5–6 °C higher melting temperatures relative to wt p53, should present light scattering and ThT binding intensities between wt p53 and p73, suggesting that further stabilization of the p53 HM DBD by an additional ~2–3 °C might diminish its aggregation to a level on par with p73.

Although p63 and p73 aggregated significantly less than p53, both light scattering and ThT results show that p73 aggregation was not negligible, as was observed for p63. Whether the relatively low amounts of p73 aggregation observed here is physiologically important remains to be seen. It has been shown that small amounts of p63 and p73 can coaggregate with several p53 mutants *in vitro*, as well as tumor cell lines and cancer tissues^10^,^27^, which may explain how mutant p53 can inhibit p63 and p73 functions^35^,^36^. While we detected non-negligible p73 aggregation in assays performed at 37 °C, structural studies of the p73 DBD using concentrated samples of 0.2–0.3 mM showed long term stability at 25 °C^37^, suggesting that aggregation is largely diminished at lower temperatures. Further investigation is required to better characterize p73 aggregation and to assess the biological effects, if any, of p73 aggregates.

The strong correlation between DBD stability and aggregation propensity provides some basis for rationalizing the differential aggregation, but does not reveal the underlying molecular features. Computational methods are indispensable tools for studying precise molecular details, which can be difficult to assess experimentally^38^,^39^. MD simulations have been employed to investigate molecular-level details of p53, such as the different physical properties of wt p53 and cancer associated mutants, as well as binding sites and mechanisms of potential therapeutic molecules^40^,^41^. In the present study, long timescale MD simulations were employed in combination with experiments to compare the structural and dynamic properties of p53 family DBDs. The simulations are at least 100 times longer than similar comparative studies^18^,^42^, providing considerably more time for conformational sampling, with all simulations performed in duplicate to verify consistency in the results.

Per-residue comparisons of structural deviation and BHB protection revealed clear differences between p53 and p63/p73 (Figs 4 and 5). Observation of structural deviation around residues 220–230 of p53 was interesting, as the region involves the mutation-prone Y220 position. The Y220C mutation is highly destabilizing and exhibits accelerated aggregation compared to full-length wt p53^10^,^43^. The simulations revealed that the region of p53 spanning 220–230 deviated considerably more from its initial structure compared to the corresponding region in p63 and p73. Based on our results, higher structural deviation in this region may be explained by deficiencies in BHB protection (Fig. 5b).

BHBs that are inadequately shielded by sidechain nonpolar groups are sites of structural vulnerability, because they are susceptible to water attack, which weakens a proteins intramolecular hydrogen bonds^44^. Underprotected BHBs, commonly referred to as dehydrons, are sticky sites that are often involved in protein associations. For instance, the p53 DBD contains groups of underprotected BHBs at its dimer interface and around residues involved in DNA and protein recognition, which become more protected upon complexation^45^. Protection of vulnerable BHBs with small molecules has also been demonstrated, with the concept becoming an emerging strategy in drug discovery and design^46^. Targeting of dehydrons induced by the Y220C mutation in the p53 DBD with small molecules containing groups capable of wrapping the labile bonds can diminish aggregation^11^,^47^. Although dehydrons can have functional roles in protein-protein interactions, they can also be promoters of amyloid aggregation^48^. The details of how dehydrons promote amyloid formation are not perfectly clear, but there is an obvious correlation between the number of underwrapped BHBs in a protein and aggregation propensity^49^. One possible explanation is that although folded proteins normally adopt specific tertiary structures based on specific sidechain contacts, under some conditions, main chain interactions, which are the primary forces in amyloid formation, become dominant^50^,^51^. However, proteins with very high percentages of underprotected BHBs, such as prion, can convert to amyloid under physiological conditions^31^.

Our data suggests that, even in absence of mutation, the region encompassing residues 220–230 of p53 is prone to structural deviation, and therefore exists in a state that is primed for further destabilization when key residues located in this region are mutated. The innate structural weakness of this region is likely a complicating factor for targeting the site in Y220C mutants with small molecule stabilizers. Despite considerable efforts in finding high-affinity molecules, dissociation constants of the best candidates are still >10 μM^40^. The other region where p53 showed higher structural deviation compared to p63/p73 was the S6/S7 turn (residues 207–213). A recent computational study also found structural variation in this region^41^, but the molecular basis for defect formation are still unclear. While the region of deviation around 220–230 overlapped a patch of underprotected BHBs in the same area, BHB protection around 207–213 was comparable to that of p63. Therefore, it was difficult to establish if the S6/S7 defect is related to BHB protection, but the possibility that it is associated a nearby region of exceptionally low BHB protection cannot be ruled out (Fig. 5c, label 2). This region encompasses H1, which forms a major part of the dimerization interface. Homodimerization of p53 is favorable, but occurs with low probability for p63 and p73^52^. Because dehydrons are promoters of molecular association, higher dehydron density in H1 may explain why p53 is more prone to dimerize. However, prior to dimerization, the dehydron-rich site likely promotes structural weakness, especially because it forms steric contacts with other segments of lower BHB protection.

The HHP-induced mechanism of pushing water molecules into the protein structure^23^,^24^, together with the presence of exposed BHBs in p53 segments is a reasonable explanation for the lower stability of p53 in comparison to p63 and p73 against pressure. Hydration of these identified segments would represent nucleating sites for protein unfolding, and ultimately aggregation of p53. Even using the superstable p53 QM DBD, hydration effects triggered by pressure were not avoided to the same extent as p63/p73. Thus, a higher degree of underprotected BHBs correlates with lower stability to pressure, and well-protected BHBs contribute to shielding against pressure denaturation.

Together, the RMSD and BHB analysis identify new and previously reported sites of structural vulnerability in the p53 DBD. These regions may represent sites of structural weakness where initial unfolding takes place, and are therefore crucial therapeutic targets. Our pressure experiments add a new piece of evidence for this hypothesis. It is also noteworthy that despite importance of identifying these regions, the BHB wrapping results were not obvious based on static structures (similar to the finding reported in^45^), illustrating the importance of dynamics to unveil molecular detail. The BHB protection patterns reported here provide a basis for evaluating the efficacies of engineered higher stability p53 DBD constructs and identifying sites of structural vulnerability induced by disease-related p53 mutations.

In addition to our major findings, there were several additional notable results related to N-terminal extension in p53, p53 R175H mutation, and potentially important inter-residue contacts in p73. Although the N-terminal extended p53 DBD was found to have more native contacts, and considerably reduced structural deviation compared to the shorter p53 construct (Supplementary Table 1 and Fig. 4a), it still exhibited the major regions of higher RMSD and BHB underprotection, compared to p63 and p73 (Figs 4a and 5b). The persistence of these sites of structural vulnerability explain why p53 Ext is still aggregation prone^21^. MD simulations of p53 R175H were highly consistent with those of wt p53, despite experimental data indicating considerable structural differences^53^. The contrasting results can be explained by the fact that in the simulations the structural Zn^2+^ was not allowed to dissociate. For proper folding, the p53 DBD requires a single Zn^2+^ that binds close to R175^53^. Prior work shows that the R175H mutant exhibits lower zinc affinity, and more rapid zinc loss than wt p53 DBD, resulting in a higher population of unfolded molecules^53^,^54^. Therefore, although the consequences of R175H mutation on DBD structure were not seen in the simulations, the data supports therapeutic approaches that aim to prevent of zinc dissociation as a means to reactivate p53 mutants impaired in zinc binding^54^. Another interesting finding was the identification of an amino acid substitution that may be important for p73 stability or function. In p73, the D189-R269 residue pair, which corresponds to E171-R249 in p53, was distanced too far apart to form electrostatic interactions (Fig. 4e), presumably due to the shorter sidechain. While the E171-R249 interaction is structurally important for p53^28^,^55^, further studies are required to assess the stability effects of the substitution in p73. The sequence difference could also have functional effects. Being located near the DNA binding surface, it could play a role in regulating binding to different gene targets.

In summary, we show that the wt p53 DBD is considerably more aggregation prone compared to p63 and p73 DBDs, which exhibited negligible and slow aggregation, respectively. MD simulations indicated specific regions of p53 that deviate substantially more from the initial structure, and showed elevated incidence of underprotected BHBs compared to p63 and p73. The results point to regions of structural weakness that may be prone to unfolding, and hence indicate new targetable sites for modulating p53 stability and aggregation.

There are some other interesting strategies targeting p53 aggregation that are worthy to comment on. Here, and in several other works, the aim is to push the equilibrium towards stable, folded conformations, thereby lowering the population of unstable, or poorly folded molecules, which are the ones prone to aggregate. Other strategies target aggregation. Soragni *et al*.^56^ show that a peptide containing the aggregation nucleus sequence, with a single arginine substitution at position I254, binds to the same region of unstable p53 molecules exposing their nucleus, preventing further aggregation. The peptide is based on findings by Xu *et al*.^27^ showing that full-length p53 with the I254R mutation does not aggregate in cells. Despite encouraging results using I254R substituted peptides to avoid p53 aggregation, Lei *et al*.^57^ caution against its use due to favorable cross-interaction between wt and arginine substituted species that shift otherwise disordered I254R peptides into an aggregating state. Other approaches being investigated for targeting aggregates of p53 and other amyloids include use of modified peptides, molecular tweezers, and small molecule polyphenols, such as curcumin and resveratrol^58^. An important consideration for application of the aforementioned strategies for p53 is the unique nature of p53 aggregation. Compared to typical amyloids, such as amyloid-*β* and α-synuclein, p53 aggregation does not follow a slow nucleation model^11^. Instead, p53 aggregates rapidly, showing little or no lag phase (Fig. 1). Because many strategies for targeting amyloid aggregation aim to disrupt the initial nucleation events, such as preassembly of small oligomers, it is necessary to assess the efficacies of these approaches on p53 aggregates.

## Methods

### Protein expression and purification

cDNA of His6 tagged human proteins (p53 DBD 94–312 Addgene 24866, p53 QM DBD, p63 DBD 166–362, and p73 DBD 112–311 Addgene 39077) were transformed into *E. coli* BL21 (DE3) cells, and grown at 37 °C in LB medium to an OD_600_ of ~0.7, and subsequently induced with 0.5 mM IPTG for 16 hours at 15 °C. The p53 QM construct (M133L/V203A/ N239Y/N268D) was engineered by site-directed mutagenesis of the wt p53 construct (Genscript). The p63 DBD was synthesized by GenScript (Piscataway, NJ) into pUC57 standard vector after codon optimization for *E. coli* and subcloned into pET28a vector (Novagen, San Diego, CA) using NdeI/BamHI sites to produce a N-terminal His6 tagged protein. The proteins were purified from the soluble fractions on columns of Ni-NTA Agarose (Qiagen), followed by gel filtration chromatography, as required. Purified proteins were dialyzed against 50 mM sodium phosphate pH 7, 100 mM NaCl, and 2 mM DTT.

### Aggregation assays

Aggregation was assessed using light scattering and ThT fluorescence. Light scattering experiments were performed on a K2 Multifrenquency Phase spectrofluorometer set at 500 nm for excitation and emission (excitation slit width 0.5 mm, emission slit width 0.5 mm). DBD samples of 2 μM in 50 mM sodium phosphate pH 7.0, 100 mM NaCl and 2 mM DTT were stirred continuously at 37 °C during the experiments. Detection of amyloid aggregates was performed by measuring ThT fluorescence at 480 nm upon excitation at 440 nm, using a SpectraMax Paradigm Multi-Mode Detection Platform (Molecular Devices) with temperature maintained at 37 °C. Time-resolved fluorescence was recorded immediately after adding 5 μM protein to pre-equilibrated buffer containing 20 μM ThT.

### Pressure experiments

Pressure titrations were recorded using an ISSK2 spectrofluorometer (ISS, Inc.) equipped with a high-pressure cell. Tyr/Trp emission spectra from p53 family members were collected from 1 bar to 3.1 kbar at 25 °C. Protein samples of 5 μM were left for 5 min at each pressure before excitation. Tyr/Trp residues were excited at 280 nm using a slit width of 1 mm, and emission was recorded from 290 to 400 nm using a slit width of 2 mm and step-size of 2 nm. Concomitant light scattering measurements were taken after each pressure increment with excitation at 320 nm and emission from 300 to 340 nm (slit width of emission were reduced to 0.5 mm). Changes in Tyr/Trp emission spectra were quantified as the center of spectral mass, CM = ∑*v*_*i*_*F*_*i*_/∑*F*_*i*_ where *F*_*i*_ stands for fluorescence emitted at wavenumber *v*_*i*_ and the summation is carried out over the range of appreciable values of *F*, and subtracted to the value at 1 bar (Δ center of mass, cm^−1^). Increased light scattering against pressure is shown as the area under the scattering plot. Protein buffers were changed to 50 mM Tris-HCl pH 7.4, 150 mM NaCl and 5 mM DTT using 10,000 MWCO Amicon Ultra-15 centrifuge filter units (Millipore). Experiments were performed three times with different protein preparations and results are expressed as the avg. ± s.e.

### MD simulations

Structures for the MD simulations were obtained from the PDB (p53–2FEJ, p63–2RMN, p73–2XWC, and p53 Ext–2XWR)^21^,^30^,^33^,^57^. The p53 R175H mutant was generated from the p53 structure using Chimera software^59^. A short loop (residues 265–267) missing from the p73 DBD structure was reconstructed with FALC-Loop^60^. p53, p63 and p73 structures spanned exactly the amino acid ranges in Fig. 3a, while the p53 Ext structure spanned residues 91–289. Acetyl and NH_2_ capping groups were modeled onto the N and C termini, respectively, of all starting structures. For consistency, protonation states of ionizable side chains were assigned identically for all structures, based on their most probable form at pH 7. Histidine residues were protonated on ND1 only, except for the Zn^2+^-coordinating histidine (position 179 in p53), which was protonated on NE2 only. Cysteines involved in Zn^2+^ coordination (p53 positions 176, 238, and 242) were deprotonated. The DBDs bind a structurally important Zn^2+^, which was maintained at its native location using distance restraints between the ion and coordinating residues to avoid the possibility of dissociation. Structures were centered in cubic boxes of side length 7 nm and solvated with TIP3P water^61^. A charge-neutral system and physiological salt concentration of 0.15 M was achieved by addition of Na^+^ and Cl^−^. AMBER99SB*-ILDN parameters were used for all system components, and simulations were carried out with GROMACS 4 software^62^ using periodic boundary conditions. Steepest descents energy minimization was performed, followed by 2.5 μs production simulations. Duplicate simulations of 2.5 μs were performed for each system, with the only difference being the assignment of different initial atom velocities. In total, 5 μs of simulation was performed for each system. Simulations were performed integrating the equations of motion with a 2 fs timestep in the constant particle number, pressure and temperature (NpT) ensemble. Temperature was set at 310 K, and controlled by the Parrinello-Donadio-Bussi^63^ v-rescale algorithm with a coupling time constant of 0.1 ps. Pressure was set at 1 bar, and controlled by the Parrinello-Rahman^64^ barostat with a coupling time constant of 0.5 ps. Lennard-Jones and short-range electrostatic interactions were cut off at 1.0 nm. Long-range electrostatics were handled with Particle-Mesh Ewald^65^.

### Simulation analysis

To facilitate comparison of corresponding residues in the different family members, residue number labeling used in figures and axes references p53 positions. Due to p63 and p73 containing 2 additional residues (Fig. 3a), data values for these residues were removed to maintain consistent residue numbering. Hydrogen bonds were detected using an acceptor-donor distance cutoff of 0.35 nm, and an acceptor-donor-hydrogen angle cutoff of 30°. Protection analysis of hydrogen bonds was carried out similarly to previously described methodology^66^. The number of protecting groups (wrappers) per hydrogen bond were quantified by counting the number of unique nonpolar carbon atoms within the desolvation domain, defined by two spheres of radius 0.65 nm centered at the Cα atoms of the acceptor-donor pair. Because wrapper quantification is sensitive to parameters used for hydrogen bond detection and especially the desolvation domain radius, a set of 500 high-resolution PDB structures (Top 500 list)^32^ was evaluated with the parameters used in this study. The resulting distribution had a mean of 26.8 and standard deviation of 8.2, which is the same as obtained in prior wrapping analysis of large numbers of high quality PDB structures using similar hydrogen bond geometric criteria and desolvation domain radius (http://www.pymolwiki.org/index.php/Dehydron) and ref. 67. Therefore, the standard definition for underwrapped hydrogen bonds (those with less than 19 wrappers; 1 standard deviation below the mean) was appropriately used here.

## Additional Information

**How to cite this article**: Cino, E. A. *et al*. Aggregation tendencies in the p53 family are modulated by backbone hydrogen bonds. *Sci. Rep*. **6**, 32535; doi: 10.1038/srep32535 (2016).

## Supplementary Material

Supplementary Information
