# Supplementary material for: Aggregation tendencies in the p53 family are modulated by backbone hydrogen bonds
Source: Sci Rep. 2016 Sep 7;6:32535. doi: 10.1038/srep32535 (PMC5013286; doi:10.1038/srep32535)
Supplement: Supplementary Information [file srep32535-s1.pdf]

Aggregation tendencies in the p53 family are modulated by backbone hydrogen bonds

Elio A. Cino*, Iaci N. Soares, Murilo M. Pedrote, Guilherme A.P. de Oliveira, and Jerson L. Silva

*Elio A. Cino, Programa de Biologia Estrutural, Instituto de Bioquímica Médica Leopoldo de Meis, Instituto Nacional de Biologia Estrutural e Bioimagem, Centro Nacional de Ressonância Magnética Nuclear Jiri Jonas, Universidade Federal do Rio de Janeiro, 21941-902, Rio de Janeiro, RJ, Brasil. E-mail: eliocino@gmail.com

Supplementary Table 1. Comparison of parameters from MD simulations[a].

	p53	p63	p73	p53 R175H	p53 Ext
Cα RMSD[b] (nm)	0.27 ± 0.01	0.22 ± 0.02	0.24 ± 0.01	0.27 ± 0.04	0.18 ± 0.02
Fraction native Cα contacts[c]	0.60 ± 0.02	0.68 ± 0.02	0.69 ± 0.04	0.61 ± 0.03	0.78 ± 0.04
Secondary structure[b,d]	0.60 ± 0.03	0.57 ± 0.03	0.58 ± 0.03	0.60 ± 0.03	0.57 ± 0.03
Radius of gyration (nm)	1.64 ± 0.01	1.65 ± 0.01	1.66 ± 0.01	1.63 ± 0.01	1.63 ± 0.01
Number of Cα-Cα contacts within 1.0 nm[b]	8.4 ± 0.1	8.2 ± 0.1	8.2 ± 0.02	8.3 ± 0.004	8.4 ± 0.05
Intramolecular backbone hydrogen bonds[b]	0.41 ± 0.01	0.40 ± 0.002	0.41 ± 0.001	0.41 ± 0.001	0.39 ± 0.01
Total intramolecular hydrogen bonds[b]	0.74 ± 0.004	0.69 ± 0.01	0.70 ± 0.004	0.71 ± 0.01	0.73 ± 0.004
Protein-water hydrogen bonds[b]	2.2 ± 0.05	2.2 ± 0.05	2.2 ± 0.01	2.2 ± 0.004	2.1 ± 0.01

[a]Values reported correspond to avg. ± s.d. between the two replicates.
[b]Values normalized per residue.
[c]Cα deviations <0.15 nm from the initial structure were classified as native contacts.
[d]Sum of residues in α-helix, β-sheet, β-bridge, and turn conformations, as classified by DSSP.
